# An Investigation of a Biomimetic Optical System and an Evaluation Model for the Qualitative Analysis of Laser Interference Visual Levels

**DOI:** 10.3390/biomimetics9040220

**Published:** 2024-04-07

**Authors:** Jin Niu, Xiping Xu, Yue Pan, Zhenhao Duan

**Affiliations:** School of Opto-Electronic Engineering, Changchun University of Science and Technology, Changchun 130022, China

**Keywords:** bionic optics, laser-induced visual interference, quantitative evaluation, grey analytic hierarchy process, photoelectric detection

## Abstract

To objectively quantify the level of visual interference induced by lasers, we developed a biomimetic optical system designed to emulate human vision. This system is based on an optical model of the eye and synthetic imaging principles, allowing it to generate biomimetic optical images that closely mimic human visual perception. Upon exposure to a 532 nm laser, biomimetic optical images were captured under various ambient lighting conditions. By employing a contrast threshold model for human visual target detection and grayscale hierarchy analysis, we devised an evaluation model to quantify the levels of laser-induced visual interference. The bionic images obtained from our experiments, in conjunction with the constructed model, enabled us to assess the degree of laser-induced visual interference. Our results indicate that this system can effectively substitute the human eye when testing laser imaging effects, with the generated bionic images achieving up to 90% concordance with human vision. The proposed evaluation model facilitates the quantitative analysis of laser-induced visual impairment. This apparatus and evaluation model hold significant promise for the precise quantification of laser-induced visual interference levels.

## 1. Introduction

Lasers are extensively utilized across various sectors, including industry, medicine, and research. However, concerns regarding visual safety persist due to the potential hazards posed by even low-energy laser beams, which can result in glare, temporary blindness, or vision impairment [1,2,3,4,5]. The increasing incidence of laser-related safety incidents has prompted global efforts to develop and investigate the extent of laser interference with vision. This necessitates the use of lower-power lasers to ensure safer and more effective outcomes during laser applications [6]. Therefore, it is crucial to possess the capability to quantitatively, reproducibly, and accurately assess the degree of visual interference caused by lasers. This is essential for ensuring the safe use of laser sources and minimizing the risk of serious injury. Given that vision is a subjective sensory experience, determining how to quantify it into objective data through bionic simulation poses an urgent challenge.

Currently, numerous methods exist for quantifying the extent of laser-induced damage to the human eye. However, in terms of examining the impact of lasers on vision, much research remains limited by the test parameters considered. Researchers have conducted extensive experimental studies that can be broadly categorized into biological and non-biological tests. Biological assessments include visual electrophysiological examinations, monitoring retinal laser energy density, and tracking the retinal signal recovery time following flicker stimulation in animals such as rhesus monkeys and blue rabbits. These are followed by a graded evaluation of the effects’ severity, with key parameters like laser energy density, recovery time, and degree of physiological impairment being recorded [7,8,9,10,11,12]. Most animal studies selected laser power densities exceeding 100 mW/cm^2^, radiation distances less than 10 m, and exposure durations longer than 1 s, limiting their applicability across various viewing conditions and ambient light levels. Human testing is costly and time-consuming and requires ethical approval, which may also restrict the ability to perform repeated testing and thoroughly explore the parameter space. Moreover, due to inevitable risks, exposures beyond established safety thresholds cannot be investigated for visual effects in human subjects. Even at “safe” exposure levels, the long-term health implications of frequent exposures remain poorly understood. Consequently, abiotic approaches are becoming increasingly prevalent, overcoming these limitations by mimicking the structure of the human eye and displaying the results on a computer screen for the safe observation of effects. Drs. Craig Williamson and Leon McLin conducted extensive research on how lasers interfere with vision [13,14,15] and developed a glare simulator. One limitation of this simulator is its reliance on rectangular detectors in ZEMAX, which have uniformly distributed “pixels”, unlike the variable density photoreceptors in the retina. Furthermore, while the simulation model in the visualization tool developed by Williamson can accurately simulate the glare effect in various situations, it may not be suitable for studies involving different optical components or for simulating a specific real-world scenario with all relevant parameters. Therefore, further validation and refinement will be necessary in the future.

To address the aforementioned challenges, we developed a biomimetic optical system capable of quantitatively analyzing the extent of visual interference induced by lasers. This system is specifically designed for practical laser applications and generates laser images that accurately replicate visual effects. To assess the visual impact, we constructed an assessment model by integrating the contrast threshold model for human eye target recognition with the gray-level analytic hierarchy process. The efficacy of this system was validated through imaging tests, data processing, and effect evaluation on a representative 532 nm laser.

## 2. Biomimetic Eye Optical System

### 2.1. System Components and Functions

**Figure 1:**
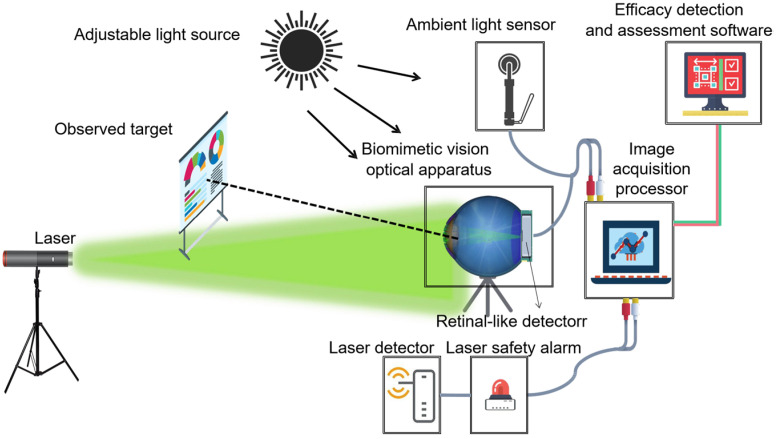
The biomimetic optical system.

As depicted in Figure 1, the biomimetic optical system was specifically engineered to quantify and analyze pertinent parameters associated with laser visual interference in designated scenarios. In essence, it can serve as a surrogate for the human eye and provide a real-time display of the biomimetic imagery of laser visual interference within the current environment. The system comprises a biomimetic vision optical apparatus, an ambient light sensor, a laser detector, a laser safety alarm, an image acquisition processor, and efficacy detection and assessment software.

The biomimetic vision optical apparatus is composed of simulated human eye optical components, a retina-like detector, and an artificial eyeball support structure that are used to capture and output bionic human eye laser raw images.

The ambient light sensor measures the luminance at the target location, providing these data to the image acquisition processor. Its model number is CMI-200 and its measurable brightness range is from 1 × 10^−4^ cd/m^2^ to 1 × 10^3^ cd/m^2^.

The image acquisition processor is a computer-based system capable of real-time data collection and processing, performing bionic optimization on the laser raw images.

The laser detector ascertains the peak laser wavelength and power density at the pupil. This detector can detect laser sources within a working wavelength band of 380 nm to 780 nm, with a spectral resolution of 1 nm.

The laser safety alarm determines if the detected value surpasses a preset safety threshold and is situated at the data collection end of the laser detector. To safeguard test personnel and the testing system from potential laser damage during the research phase, preliminary information about the laser source is initially entered into the laser safety alarm for assessment. Only when the laser power does not exceed the specified safety threshold will the simulation of the human eye target be activated for testing, with the laser source information parameters concurrently provided to the image acquisition processor for subsequent processing. The safety threshold for the laser power follows relevant standards [16] and is set by the tester with a weight value typically between 0.3 and 0.8.

### 2.2. System Design

#### 2.2.1. Biomimetic Eye Optical Model Design

The human eye represents one of the most intricate organs within the anatomical structure. Although it may initially seem like a simple refractive system, the states of its various components alter when observing objects at different distances and under varying lighting conditions. Additionally, the human eye undergoes changes with age and varies among individuals. Laser light scattering within the eye significantly disrupts normal visual perception. Therefore, an optical model of the human eye designed to investigate the extent of visual disruption caused by lasers must be crafted. By constructing an optical architecture focused on imaging under laser illumination, the developed laser-interfered vision eye model could simulate and enhance the capture of laser speckles at the retina.

**Figure 2:**
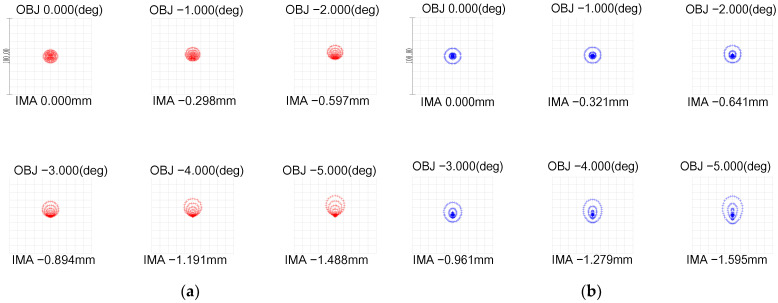
Comparison of retinal diffuse spot images from the two models: (**a**) Gullstrand exact eye model; (**b**) laser interference visual eye model.

**Figure 3:**
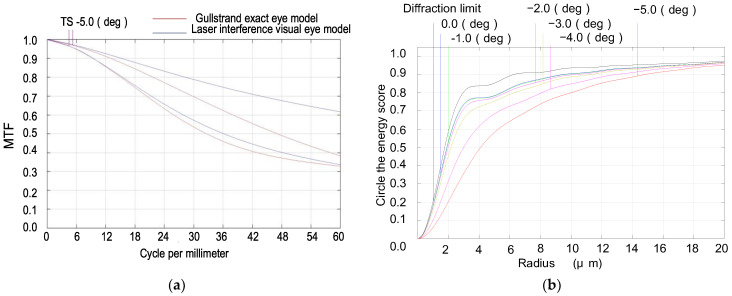
MTF comparison and diffraction circle energy ratio comparison of the two models: (**a**) MTF comparison; (**b**) diffraction circle energy ratio comparison.

The Gullstrand exact eye model is widely recognized as the most authoritative and closely mirrors the actual refractive condition of the human eye [17]. It is generally acknowledged that this model epitomizes the standard level of aberration for the human eye in a non-accommodative state, particularly concerning image quality. Upon immediate laser exposure, individuals naturally perform avoidance maneuvers, such as blinking or turning their heads. As a result, during these brief laser exposure instances, the human eye remains in a non-accommodative state. Based on Gullstrand’s exact eye model, we thus refined and developed a laser interference visual eye model. This model comprises six refractive surfaces: the anterior and posterior surfaces of the cornea, the anterior and posterior surfaces of the lens cortex, and the anterior and posterior surfaces of the lens nucleus. The refractive attributes of this model are consistent with those of the actual human eye’s refractive structures, and its performance approximates that of the human eye. Several optimizations were performed to ensure a close resemblance to the authentic human eye:

1. To preserve the imaging quality of the lens without compromising the detector’s capability, we replaced the glass material with a combination of MGF2-E, H-FK95N, H-QK3L, and H-FK95N. This maintained the relative refractive index difference between adjacent materials such that the deviation from the relative refractive index difference between any two adjacent layers (cornea and aqueous humor, aqueous humor and lens, lens and vitreous body) in the Gullstrand exact eye model was no greater than 1%.
2. With an incident pupil diameter of 3 mm (consistent with the normal human eye’s pupil diameter), we optimized the radius of the scattered spot within the paraxial region from −5° to 0° by adjusting structural parameters like curvature radii and glass spacing. Our design values deviated by no more than 10% from those in the Gullstrand exact eye model, as shown in Figure 2 and Table 1.
3. Utilizing ZEMAX design software’s operands such as EFFL, AXCL, LACL, SPHA, FCUR, and DIST, we appropriately retained the inherent aberrations of the simulated human eye optical system. As depicted in Figure 3, the MTF (Modulation Transfer Function) of our laser interference visual eye model at a −5° angle of view is 0.32@60lp/mm, with 85.5% of the energy concentrated within a 12 μm radius circle around the centroid. The MTF value for near paraxial light in this model deviates by no more than 0.1 from that of the Gullstrand exact eye model.
4. To maximize practical manufacturability, we opted for spherical lenses over aspherical designs. We also adjusted the distance between the aperture and the sixth surface to replicate the effects achieved with aspherical models. Balancing and modifying the thickness of individual lenses mitigated specific imaging discrepancies resulting from material dissimilarities between lenses and human eye tissues.

Based on the optical design outcomes of the laser interference visual eye model, we carefully selected materials and established tolerance settings to design and fabricate an optical component structure that mimicked the human eye.

#### 2.2.2. The Retina-like Detector Design

**Figure 4:**
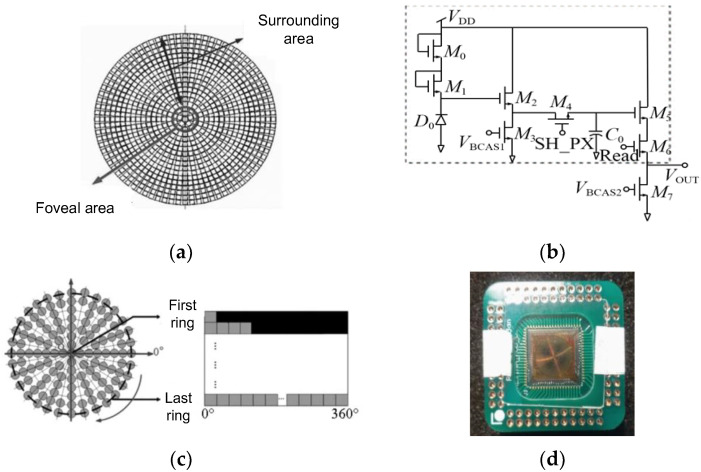
The retina-like CMOS detector: (**a**) distribution of pixel arrangements; (**b**) 7T logarithmic pixel circuit; (**c**) output matrix image; (**d**) physical layout.

In the biomimetic optical system, the image sensor functions in a manner analogous to the retina within the eye. It detects light signals that are transmitted through the lens and relays them to the subsequent image-processing system. The density of photoreceptor cells in the human retina decreases with increasing distance from the fovea, leading to non-uniform characteristics in visual information acquisition [18]. To ensure the precise analysis of imaging spots on the retina upon exposure to laser radiation, we selected an image sensor that emulated the actual structure of the retina. As depicted in Figure 4, this image sensor was a CMOS detector arranged in a ring configuration with centrally symmetrical pixels, dividing the photosensitive area into a central fovea region and a peripheral region. Considering the physiological structure of the human retina, as well as the current technological capabilities and the risks associated with the design and manufacturing process of chips featuring irregular pixel arrangements, a design was conceived that incorporated a central fovea region comprising 50 concentric rings, surrounded by a peripheral area encompassing 88 rings. This configuration resulted in a total of 138 rings for the entire detector. Table 2 presents the fundamental parameters of this detector. The variable R denotes the ratio of the maximum to minimum pixel size, which reflects the extent of pixel size variation across the detector array; Q signifies the ratio of the overall detector size to the minimum pixel size, thereby describing the field of view under conditions of equivalent data volume. The pixels utilize a 7T logarithmic pixel circuit structure, which mimics the logarithmic response of the human eye to light intensity [19,20]. This design enhances its capability to simulate the human retina’s acquisition of image information from a given scene, including images of laser spots and observation targets post-laser illumination.

#### 2.2.3. Biomimetic Processing of Laser Spot Images

The biomimetic optical model achieved significant simulation effects regarding the structure and imaging characteristics of the human eye. To study the actual impact of lasers on vision, it is essential to consider the scattering effects of various ocular components as well as non-imaging factors such as physiological variances within the human eye, environmental conditions, and contextual features of the action scene. Consequently, the refinement of initial image bionics is crucial. This refinement aims to minimize discrepancies between the test image produced by machine vision and the image perceived through human vision [21,22]. Laser image biomimicry primarily encompasses three stages: primary data acquisition, target extraction from the original image, and biomimetic emulation of the light spot.

**Figure 5:**
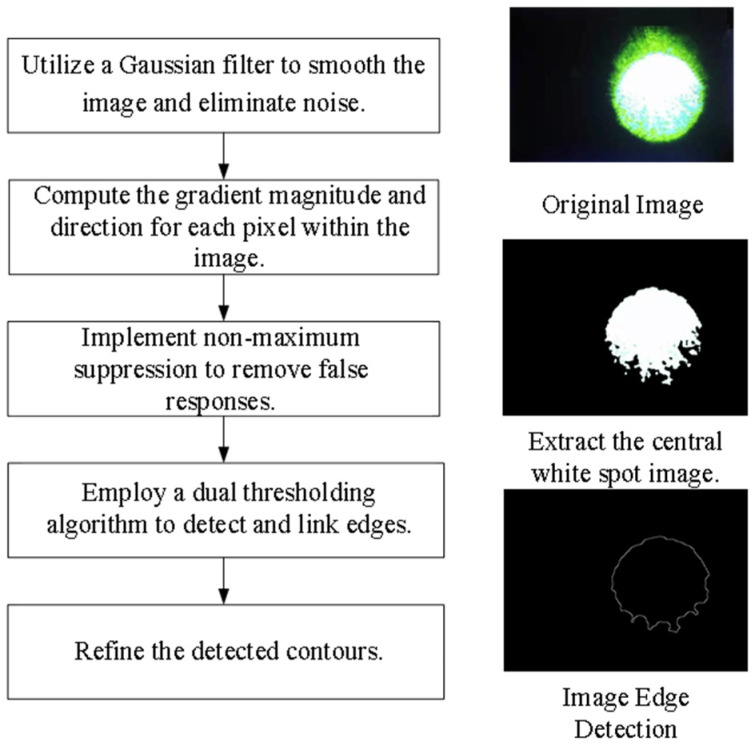
Process of detecting the edges of light spots.

1. Primary Data Acquisition. To appraise the degree of visual disturbance induced by lasers impartially, the following primary data must be gathered: technical specifications of the laser source, individual ocular parameters of the subject, contextual elements, and the unaltered image of the retinal imaging light spot produced by the biomimetic eye. The central wavelength and energy output of the laser are detected by the laser sensor and entered into the testing software. Parameters such as operational distance and individual ocular variances can either be inputted manually by the examiner or selected from default settings, ambient lighting conditions are monitored by the ambient light sensor and fed into the testing software, and the pristine image of the light spot is captured by the biomimetic optical apparatus and transferred to an image processor.
2. Target Extraction from the Original Image. As the retina-like detector gathers real-time images of the original laser light spot, these images undergo binarization, allowing for the separation of the intended light spot from its environmental backdrop based on contrasting grayscale values. The isolation of the targeted light spot is executed with the Canny arithmetic [23], enabling continuous edge detection. The extraction procedure and corresponding light spot manifestation are illustrated in Figure 5. Concurrently, the calculation of the denoised laser light spot’s radius and centroid is finalized, guiding the ensuing biomimetic emulation of the laser light spot.
3. Light Spot Bionic Fitting. We optimized the original image using the simplified human visual “Laser Glare Model” developed by Dr. Craig A. Williamson [24]. This model is based on the internationally recognized CIE disability glare standard equation [25], which intuitively illustrates the impact of laser glare on human vision, as shown in Formula (1).
  (1) $$g_{eye}(\theta ,A,p)=\frac{10}{\theta^{3}}+[\frac{5}{\theta^{2}}+\frac{0.1p}{\theta }][1+{\left(\frac{A}{62.5}\right)}^{4}]+0.0025p$$

Considering the calibration coefficients, the final formula, which represents the relationship between the luminance of the veiling luminance *f_eye_* and the radiant power density at the eye, is expressed as follows [24]:

(2) $$f_{eye}(\theta ,A,p,L_{b})=S_{1}L_{b}^{T_{1}}g_{eye}(\theta ,A,p)$$

(3) $$S_{1}=0.9147,\;T_{1}=0.1775$$

(4) $$C_{thr}\left(L_{b},a,A\right)=\Omega AF$$

(5) $$\Omega \left(L_{b},a\right)=\frac{2.6{\left(\frac{\varphi (L_{b})}{60a}+L(L_{b})\right)}^{2}}{L_{b}}$$

(6) $$\left\{\begin{array}{c} \mathrm{for}\;23<A<64,\;AF\left(A\right)=\frac{{\left(A-19\right)}^{2}}{2160}+0.99\\ \mathrm{for}\;64<A<75,\;AF\left(A\right)=\frac{{\left(A-56.6\right)}^{2}}{116.3}+1.43 \end{array}\right.$$

where *f_eye_* is the brightness of the veiling luminance, cd/m^2^; *p* is the iris pigmentation coefficient (*p* = 0 for very dark, 0.5 for dark, 1.0 for light, and 1.2 for very light eyes); *θ* is the incidence angle; *A* is the age of the observer; *L_b_* is ambient light level, cd/m^2^; V_λ_ is the eye’s photopic efficiency at wavelength λ; C = 683 Lm/W is the multiplicative constant; *C_thr_* is the threshold contrast of the human eye when identifying the target; C_orig_ is the target contrast in the absence of a laser field (non-dimensional value, the ratio of object brightness to field brightness); Ω is the calibration factor including the target angular size α and total luminance; and *AF* is the age factor.

**Figure 6:**
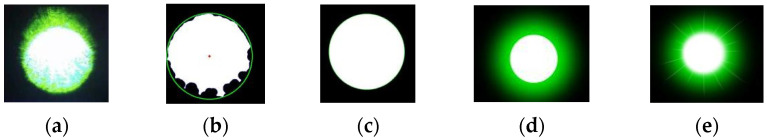
Halo biomimetic processing procedure: (**a**) original image; (**b**) center point positioning; (**c**) spot defect compensation; (**d**) biomimetic processing; (**e**) halo overlay.

Figure 6 illustrates the biomimetic fitting process for speckle patterns. Initially, defects in the integrity of the original speckle image are compensated for. The largest elliptical area within the confines of the original speckle domain is utilized to replenish the speckle pattern. A power law (gamma) transformation model is subsequently applied to enhance the contrast of the cropped image. Following this, the least squares method is employed to perform Gaussian fitting on the speckle, thus determining its central position and completing laser speckle compensation. Afterward, a biomimetic halo that mimics the actual human eye imaging effect is superimposed and fitted onto the compensated speckle. Specifically, based on the input of real test laser energy parameters and specific distribution simulations, the Gaussian distribution image section on the xy plane is obtained and integrated into the “laser interference visual model”. By inputting target human eye difference parameters and environmental variables, the biomimetic halo is derived. Then, according to the speckle radius and central position identified during initial target extraction, the simulated halo position image is optimized and fitted with the compensated speckle to produce a comprehensive speckle image.

#### 2.2.4. Simulation Eyeball Support Design

**Figure 7:**
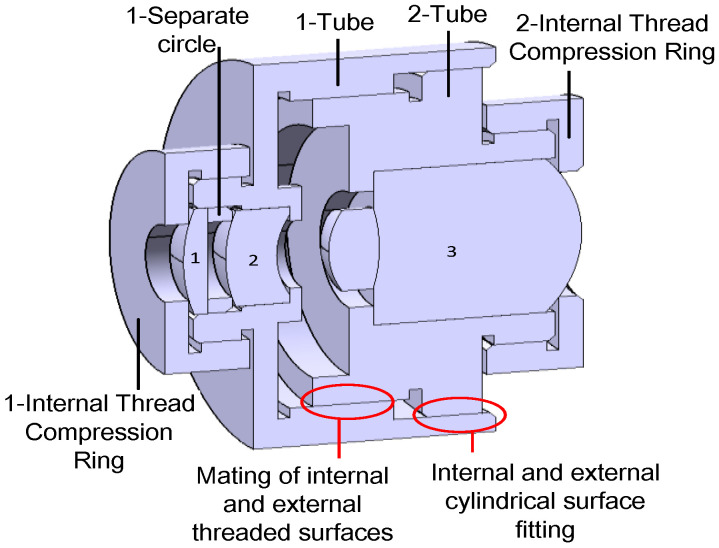
Structural diagram of the simulated eyeball support.

**Figure 8:**
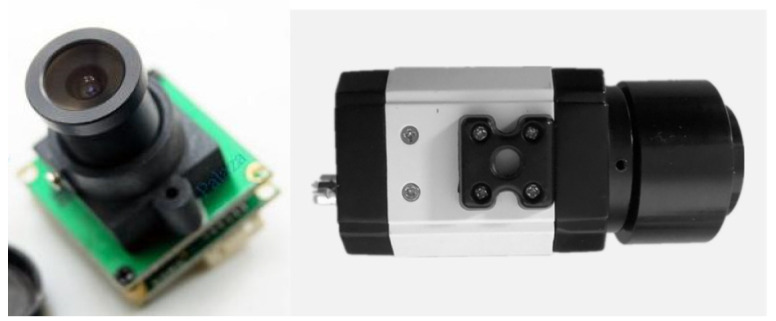
The biomimetic vision optical apparatus post-assembly.

To enhance the stability and preserve the mechanical integrity and focusing capabilities of the biomimetic eye optical device, a mechanical structural design was developed based on the outcomes of the optical design. Figure 7 illustrates this. Figure 8 shows the final assembly of the biomimetic optical device.

### 2.3. System Function Verification

**Figure 9:**
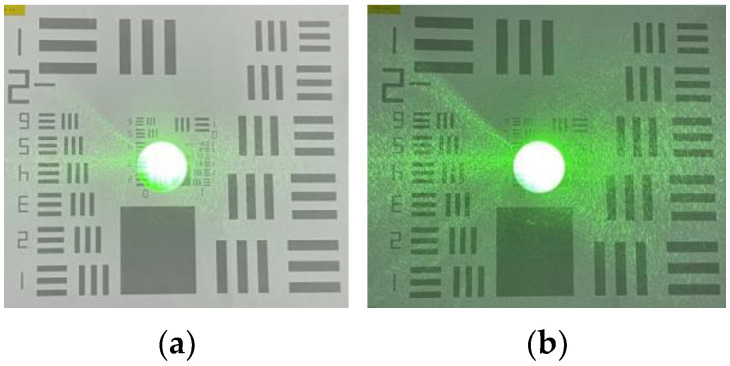
Results from the system: (**a**) bright environment; (**b**) dark environment.

We conducted a radiation test utilizing a 532 nm laser to evaluate whether the image output from our system adhered to the criteria necessary for emulating human vision. The power density of the laser, measured at a distance of 50 m, was recorded as 0.068 mW/cm^2^, which fell significantly below the maximum allowable irradiance for human eye safety standards, thus ensuring that it would not induce irreversible retinal damage in participants during the experiment. All participants participated voluntarily and obtained approval from the safety oversight committee. The efficacy of the system was assessed under two distinct lighting conditions: dim (brightness of 0.025 cd/m^2^) and bright (brightness of 235 cd/m^2^). The biomimetic optical system captured and analyzed the laser spots five times in parallel. Post data processing, the images of the biomimetic spots were displayed on the effect monitor. Figure 9 shows the imaging results of the system. Simultaneously, we solicited five volunteers of diverse ages to correlate their visual experiences with the biomimetic spot effects. The concordance rate of visual effects between the system output and volunteer feedback was then calculated. The findings revealed that upon abrupt exposure to the laser, volunteers perceived a subjective experience of a speck with varying shapes and hues manifesting in their central visual field. Gradually, the dimensions of the spot diminished, and its coloration faded until it was no longer perceptible. Initially, the speck obstructed the resolution board pattern, hindering identification; however, as the shadow’s dimensions and intensity decreased, the board’s visibility progressively improved until full discrimination was achieved, restoring the observer’s visual capacity to the pre-exposure condition. The assessment outcomes for the five volunteers are summarized in Table 3, demonstrating a visual effect matching rate of 90%.

## 3. Laser Interference Visual Level Test Evaluation Model

### 3.1. Laser Interference Vision Parameter System

**Figure 10:**
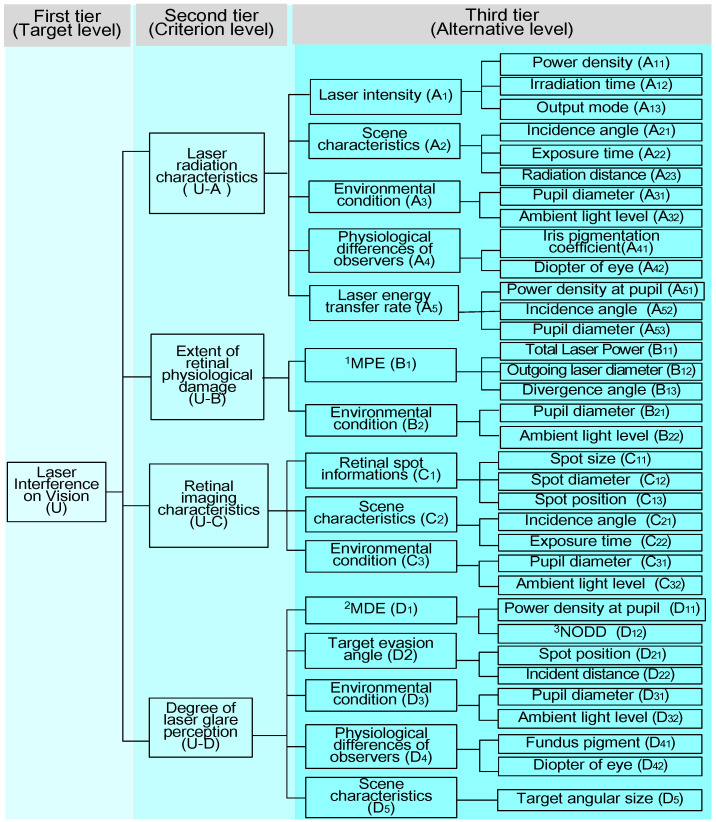
Hierarchy of laser interference visual degree assessment. ^1^ MPE, maximum permissible exposure; ^2^ MDE, maximum dazzle exposure; ^3^ NODD, nominal ocular dazzle distance [28].

The impact of lasers on vision represents a complex physiological phenomenon. The presence of a powerful laser source within the peripheral vision can significantly affect the visibility of an observed target with low contrast, thereby altering the human eye’s capability to instantaneously identify the target. Excessive laser intensity can cause physiological damage to the retina, potentially leading to psychological distress such as anxiety and stress. To quantify the level of laser interference on vision, we identified the following essential parameters: incident laser radiation characteristics (U-A), extent of retinal physiological damage (U-B), retinal imaging characteristics (U-C), and degree of laser glare perception (U-D). Each parameter comprised multiple indicators that could be specified, including laser intensity, environmental conditions, and retinal spot information. Similarly, under each level of indicators, there were factors that could further refine them. Consequently, we established a hierarchical structure for evaluating the level of laser interference vision based on the Gray Analytic Hierarchy Process (G-AHP) [26,27], as depicted in Figure 10.

### 3.2. A Comprehensive Evaluation Model Based on the G-AHP

We developed a G-AHP-based laser interference visual grading assessment model that enables experienced personnel to actively engage in the evaluation of experimental results. This is a hierarchical model wherein the first level represents the demand target of the assessment system and the second level corresponds to the criterion level. The third and fourth levels constitute the alternative level of the assessment method, as illustrated in Table 4. Quantitative indicators (A_i_, B_i_, C_i_, and D_i_) are aligned with the criteria layer, while quantitative factors (A_ij_, B_ij_, C_ij_, and D_ij_) correspond to the factor layer. Evaluation matrices (P_(U-A)_, P_(U-B)_, P_(U-C)_, and P_(U-D)_) are employed to gauge the contribution of the criterion layer. Judgment matrices (P_Ai_, P_Bi_, P_Ci_, and P_Di_) are derived from conducting specific tests with quantitative factors at the third tier.

The hierarchical evaluation can be expressed as Equations (7)–(9):

(7) $$\begin{array}{l} M_{\left(U\text{-}A\right)}=\left(\begin{array}{c} M_{\left(A_{1}\right)}\\ M_{\left(A_{2}\right)}\\ M_{\left(A_{3}\right)}\\ M_{\left(A_{4}\right)}\\ M_{\left(A_{5}\right)} \end{array}\right)P_{\left(U\text{-}A\right)}=\left(\begin{array}{c} A_{1}P_{A_{1}}\\ A_{2}P_{A_{2}}\\ A_{3}P_{A_{3}}\\ A_{4}P_{A_{4}}\\ A_{5}P_{A_{5}} \end{array}\right)P_{\left(U\text{-}A\right)};\;M_{\left(U\text{-}B\right)}=\left(\begin{array}{c} M_{B_{1}}\\ M_{B_{2}} \end{array}\right)P^{\left(U\text{-}B\right)}=\left(\begin{array}{c} B_{1}P_{B_{1}}\\ B_{2}P_{B_{2}} \end{array}\right)P_{\left(U\text{-}B\right)};\\ M_{\left(U\text{-}C\right)}=\left(\begin{array}{c} M_{C_{1}}\\ M_{C_{2}}\\ M_{C_{3}} \end{array}\right)P_{\left(U\text{-}C\right)}=\left(\begin{array}{c} C_{1}P_{\left(C_{1}\right)}\\ C_{2}P_{\left(C_{2}\right)}\\ C_{3}P_{\left(C_{3}\right)} \end{array}\right)P_{\left(U\text{-}C\right)};\;M_{\left(U\text{-}D\right)}=\left(\begin{array}{c} M_{\left(D_{1}\right)}\\ M_{\left(D_{2}\right)}\\ M_{\left(D_{3}\right)}\\ M_{\left(D_{4}\right)}\\ M_{\left(D_{5}\right)} \end{array}\right)P_{\left(U\text{-}D\right)}=\left(\begin{array}{c} D_{1}P_{D_{1}}\\ D_{2}P_{D_{2}}\\ D_{3}P_{D_{3}}\\ D_{4}P_{D_{4}}\\ D_{5}P_{D_{5}} \end{array}\right)P_{\left(U\text{-}D\right)} \end{array}$$

(8) $$\begin{array}{l} \left(\begin{array}{c} A_{1}\\ A_{2}\\ A_{3}\\ A_{4}\\ A_{5} \end{array}\right)=\left(\begin{array}{ccc} A_{11} & A_{12} & A_{13}\\ A_{21} & A_{22} & A_{23}\\ A_{31} & A_{32} & 1\\ A_{41} & A_{42} & 1\\ A_{51} & A_{52} & A_{53} \end{array}\right);\left(\begin{array}{c} B_{1}\\ B_{2} \end{array}\right)=\left(\begin{array}{ccc} B_{11} & B_{12} & B_{13}\\ B_{21} & B_{22} & 1 \end{array}\right);\\ \left(\begin{array}{c} C_{1}\\ C_{2}\\ C_{3} \end{array}\right)=\left(\begin{array}{ccc} C_{11} & C_{12} & C_{13}\\ C_{21} & C_{22} & 1\\ C_{31} & C_{32} & 1 \end{array}\right);\left(\begin{array}{c} D_{1}\\ D_{2}\\ D_{3}\\ D_{4}\\ D_{5} \end{array}\right)=\left(\begin{array}{cc} D_{11} & D_{12}\\ D_{21} & D_{22}\\ D_{31} & D_{32}\\ D_{41} & D_{42}\\ D_{51} & 1 \end{array}\right) \end{array}$$

(9) $$P_{A_{i}}=a_{A_{ij}},\;P_{B_{i}}=a_{B_{ij}},\;P_{C_{i}}=a_{C_{ij}},\;P_{D_{i}}=a_{D_{ij}};\;(i,j=1,2,\ldots ,n)$$

The classification of the initial level of laser-induced visual effects is predicated upon the secondary level of effective quantitative data, and so forth. In consideration of practical application scenarios, we further differentiated the categories of laser-induced visual effect levels. Levels I and II elicit a mild dazzling effect on vision and are not associated with therapeutic injury to the human eye. Levels III and IV exert a more pronounced influence on vision, concomitant with intense vertigo, and may inflict varying degrees of therapeutic injury to the human eye. Table 5 delineates the grayscale determination standards for the four laser-induced visual effect levels in accordance with Standard IEC 60825-1 [16].

## 4. Laser Visual Interference Test

To assess the feasibility of our system, we conducted experiments to evaluate the extent of visual interference caused by a standard 532 nm laser under varying conditions. We also analyzed the quantitative impact of specific parameters. For this purpose, we designed a comprehensive set of 21 unique scene tests labeled S-1 through S-21. The laser’s incident angle (θ) ranged from 0.5° to 12°, with a lower limit of 0.5° to prevent the obstruction of the laser beam by the target board. The laser’s radiation distance extended from 60 to 300 m. The brightness at the target location fluctuated between 0.001 and 500 cd/m^2^. The observers, whose ages ranged from 30 to 60 years with a median age of 45 years, participated in the trials. Furthermore, the study employed two distinct target contrast levels, either 89% or 40%. The laser parameters are detailed in Table 6, while the experimental parameters are documented in Table 7.

**Figure 11:**
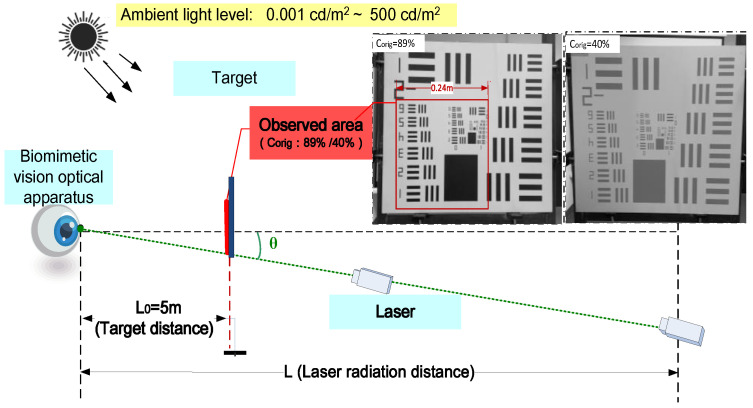
Experimental scenario layout.

The experiment was conducted in a 300 m long optical laboratory, as depicted in Figure 11. The target was positioned 5 m in front of the biomimetic vision optical apparatus. The observed area of the target measured 0.24 m by 0.24 m, corresponding to an observation angle of 2.75 degrees. The laser incidence angle, denoted by θ, was unobstructed by the target plate. The laser was positioned at a distance (L) ranging from 60 to 300 m from the biomimetic vision optical apparatus and was incident along the optical axis of the bionic eye device. Its incident light path was finely adjustable using a two-dimensional turntable. During the experiment, the biomimetic vision optical apparatus focused directly on the target area (the boxed area in Figure 11), not on the laser light source. The experimenters adjusted it to achieve clear imaging. By comparing the images captured before and after laser illumination, the angular extent of laser interference with vision was quantified.

## 5. Results

**Figure 12:**
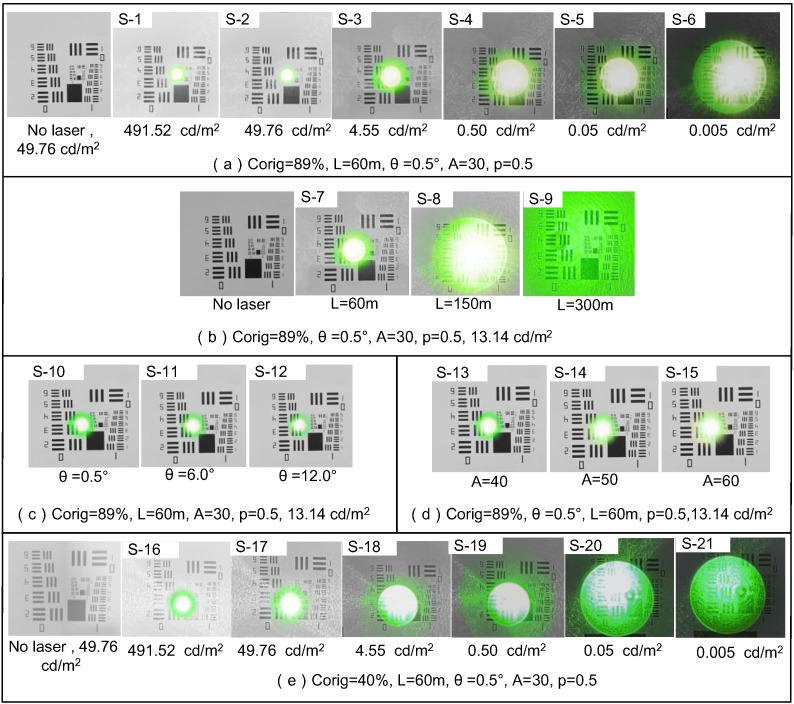
Bionic speckle images for 21 test scenarios.

When the laser power remained below the safety threshold for the human eye, the primary indicators of the extent of laser interference with vision were the target occlusion angle (U) and the transient visual effect. Biomimetic spot diagrams from 21 scenarios captured by the system are presented in Figure 12. Furthermore, the evaluation outcomes detailed in Table 8 were derived using the assessment model and subsequent data analysis.

**Figure 13:**
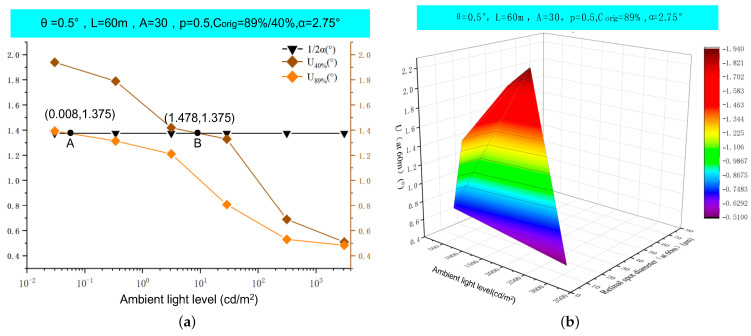
Research on the impact of ambient light levels on laser interference vision: (**a**) target evasion angle U of different contrast targets varies with ambient light level; (**b**) variation in retinal spot diameter D and target evasion angle U with ambient light level.

During our testing process, we also investigated the dose–effect relationship of the laser. Figure 13 illustrates the impact of ambient illuminance on the retinal spot diameter (D) and the target obscuration angle (U). U_40%_ represents the angular extent of the obscured portion of the target when the C_orig_ value is 40%, whereas U_89%_ represents the same for a C_orig_ value of 89%. The intersection points of U_89%_ and U_40%_ with line α are labeled as points A and B, respectively. These points correspond to scenarios in which the laser interference spot generated by the observer’s eye precisely coincides with the target area (0.24 m × 0.24 m). Notably, achieving a C_orig_ value of 89%, which resulted in complete obscurity of the target area, required an illumination level of only 0.008 cd/m^2^. In contrast, a C_orig_ value of 40% necessitated a significantly higher illumination level of 1.478 cd/m^2^. Moreover, regardless of the target contrast, both the retinal spot diameter (D) and U diminished markedly with an increase in ambient illuminance, thereby reducing the magnitude of laser-induced visual interference. Therefore, ambient illuminance has a substantial influence on the severity of visual disruption caused by the laser, particularly within the illuminance range of 0.1 cd/m^2^ to 10 cd/m^2^, where visual interference is most pronounced.

**Figure 14:**
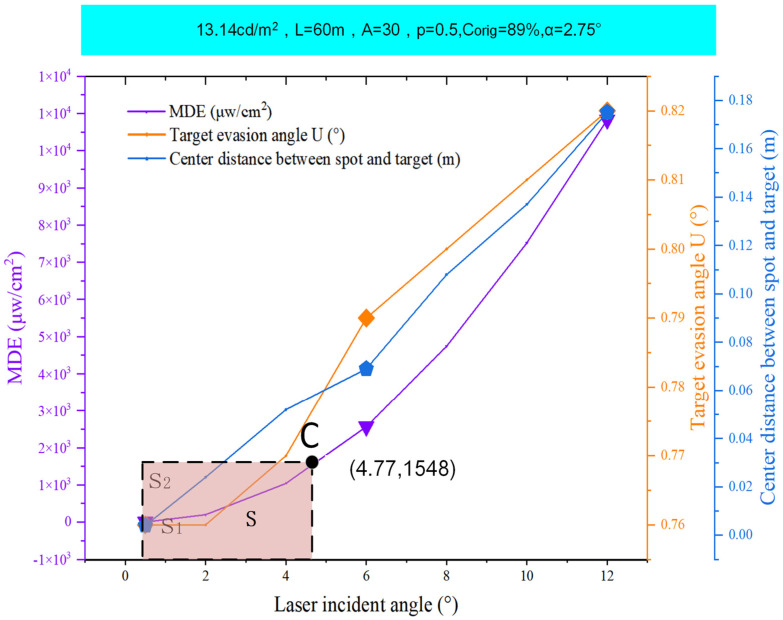
The MDE, U, and the center distance of the spot from the target on the θ change curve.

Figure 14 illustrates that as the incident angle (θ) increased, the light spot shifted away from the target’s central area, thereby mitigating the visual disturbance induced by the laser. Table 8 demonstrates that the ρ values for scenarios S-10, S-11, and S-12 were consistent at 1.537 mW/cm^2^. Point C signifies the incident angle at which the MDE value was precisely 1.537 mW/cm^2^, representing the maximum angle (θ_C_ = 4.77°) at which dizziness occurred. Between incident angles of 0.5° and 4.77°, the laser could elicit varying degrees of visual impact in the central vision area. However, when θ exceeded 4.77° up to 12°, the laser no longer exerted a visual impact in the central vision area. This finding corroborates experimental evidence from reference [28] that suggests that lasers generate visual afterimages by directly irradiating the fovea in the retina, subsequently obscuring the observed target and disrupting central vision. When a laser is employed at an oblique angle, visual afterimages may still be produced, yet they will not hinder the observed target or interfere with central vision.

According to the data collected, the deployment of this type of laser within a range of 50 to 300 m induces a visual interference level classified as II to III. The human eye possesses the capability to self-recover from this level of interference within a specified time frame, without incurring severe damage that would require medical intervention. The evaluation results are consistent with the design specifications and practical applications of the laser, corroborating the accuracy of the test outcomes and assessments derived from the biomimetic optical system and evaluation model.

## 6. Discussion

In this study, we demonstrated the efficacy of a biomimetic optical system designed for the quantitative analysis of visual interference caused by lasers. This system, modeled after the optical properties of the human eye, was validated through comparative trials and shows up to 90% concordance with the human eye’s visual perception, thereby making it a viable surrogate for human observation in the assessment of laser-induced visual interference. Additionally, an array of tests conducted across diverse scenarios and environmental conditions underscore the significance of this research for the precise quantification of laser visual interference. Looking ahead, these findings could facilitate the development of a comprehensive database encompassing wavelength, energy, and interference levels, which would serve as a guideline for the safe deployment of lasers in various domains such as medicine, manufacturing, and metrology. For instance, within the medical field, this system could enable quantitative investigations into the effects of lasers on human vision, thereby facilitating research on eye disorders and the development of novel therapeutic approaches for such conditions and enhancing ophthalmic instrumentation. In the realm of safety assessment, the system allows for the examination and evaluation of laser radiation safety with respect to the human eye, which is crucial for formulating pertinent safety standards and precautions during the development and deployment of laser technology. Moreover, within optical engineering, the system proves invaluable not only for the creation and validation of diverse visual instruments but also as an evaluative tool for vision that can be used in the testing and refinement of optical components to ensure that envisioned products meet ergonomic standards. Finally, in the domain of medical education, the system can serve as an exemplary educational tool, enabling students to achieve a comprehensive understanding of the human eye’s functionality.

Future research endeavors focusing on our system could encompass the following facets:

1. The current imaging system employs a liquid lens that more accurately replicates the scattering effect inherent to the human eye, potentially reducing the intricacy of biomimetic image processing. This enhancement could plausibly improve the system’s congruence with human visual output by an additional 3% to 5%.
2. Consideration must be given to defining the threshold range and selecting detectors with appropriate receiving wavelength ranges. It is also essential to investigate whether varying laser energies, spectra, or broad-spectrum sources induce deviations in imaging results due to photodetector interactions.
3. The individual variations in human eyes, including refractive errors, cataracts, and other ocular pathologies that contribute to differences in straylight levels, should be further investigated. These variations may affect the extent of laser-induced visual perturbations. Straylight values for study participants can be obtained during volunteer testing sessions.
4. The present study did not implement specific filtering measures for phase noise during laser spot image processing. To enhance the image quality more effectively, future research should conduct a comprehensive analysis of the noise model and devise tailored noise-filtering strategies.
5. Tailoring designs to cater to specific application scenarios would broaden the system’s applicability across diverse fields, including scientific research, medical applications, military usage, and other investigative domains.

## Figures and Tables

**Table 1:** Comparison of light spot size when the incident aperture was 3 mm.

Angle of View (°)	Speckle Size (μm)		Deviation (%)
	Laser Interference Visual Eye Model	Gullstrand Exact Eye Model	
−5	9.823	10.006	1.8%
−3	7.11	7.604	6.5%
−1	5.882	6.119	3.9%
0	5.736	5.91	2.9%

**Table 2:** Parameters of the retina-like CMOS detector.

Name	Value
Number of central rings	50
Number of central pixels	6306
Number of edge pixels	45,760
Total number of rings	138
Total number of pixels	52,066
Minimum pixel size	14 μm
R value	7.07
Q value	731
Radius of photosensitive area	5113 μm

**Table 3:** Results of comparison test.

Lighting Conditions	System	Volunteers	Age	Visual Effects Match Situation
Bright (235 cd/m^2^)	1	NO.1	25	Matched
	2	NO.2	33	Matched
	3	NO.3	35 (nearsighted)	When not wearing glasses, the match was good; when wearing glasses, the visual stimulus was slightly stronger.
	4	NO.4	42	Matched
	5	NO.5	48	Matched
Dim (0.025 cd/m^2^)	1	NO.1	25	Matched
	2	NO.2	33	Matched
	3	NO.3	35 (nearsighted)	Matched
	4	NO.4	42	Matched
	5	NO.5	48	Matched
Visual Matching Degree		90%		

**Table 4:** Evaluation model hierarchy table.

First Tier (Target Level)		Laser Interference on Vision			
Second Tier (Criterion Level)		Incident Laser Radiation Characteristics (U-A)	Extent of Retinal Physiological Damage (U-B)	Retinal Imaging Characteristics (U-C)	Degree of Laser Glare Perception (U-D)
Third Tier (Alternative Level)	Indicator layer	A_1_–A_5_	B_1_–B_2_	C_1_–C_3_	D_1_–D_5_
	Factor layer	A_11_–A_13_ A_21_–A_23_ A_31_–A_32_ A_41_–A_42_ A_51_–A_53_	B_11_–B_13_ B_21_–B_22_	C_11_–C_13_ C_21_–C_22_ C_31_–C_32_	D_11_–D_12_ D_21_–D_22_ D_31_–D_32_ D_41_–D_42_ D_51_

**Table 5:** Criteria for grayscale determination for each metric and the visual level of laser interference.

Judgment Intervals	Contribution Rate				Level	Judgment Criteria		
	Low	Moderate	High	Extremely High		Visual	Effect	Standard IEC 60825-1
E_(U-A)_	0–2	2–4	4–5	5–6	Ⅰ	No discomfort; produces less visual obscuration.	very weak	Class 1
E_(U-B)_	0–3	3–5	5–6	6–9	Ⅱ	Piercing; produces much visual obscuration. Self-healing without treatment.	weak	Class 1M
E_(U-C)_	0–2	2–3	3–4	4–5	Ⅲ	Piercing and dizziness; produces much visual obscuration. Self-healing without treatment.	moderate	Class 2
E_(U-D)_	0–2	2–3	3–4	4–5	Ⅳ	Strong piercing, dizziness, and burning sensation; produces much or complete visual obscuration. Recoverable after treatment.	strong	Class 2M

**Table 6:** Laser-specific parameters.

Laser Parameter	Center Wavelength	Average Power	Radiation Divergence	Mode	Declared Distance of Temporary Dazzle	NOHD
Value	532 nm	100 mW	(1.5 × 1.5) mrad	CW	(50–300) m	47.1 m

**Table 7:** The experimental parameters.

C_orig_ (%)		L_b_ (cd/m^2^)		θ (°)		A (Years) (*p* = 0.5)	
Scene Number	Value	Scene Number	Value	Scene Number	Value	Scene Number	Value
S-1~S-15	89	S-1,S-16	0.005	S-1~S-10, S-13~S-21	0.5°	S-1~S-12, S-16~S-21	30
		S-2,S-17	0.05				
		S-3,S-18	0.50				
S-16~S-15	40	S-4,S-19	4.55	S-11	6.0°	S-13	40
		S-5,S-20	49.76			S-14	50
		S-6,S-21	491.52	S-12	12.0°	S-15	60
		S-7~S-15	13.14				

**Table 8:** Primary test data for parameter groups for 21 scenarios.

Parameter	D (μm)	P (mW/cm^2^)	U (°)	Level
S-1	70.23	1.537	1.62	II
S-2	54.15	1.537	1.52	II
S-3	45.67	1.537	1.39	III
S-4	23.63	1.537	0.88	III
S-5	11.04	1.537	0.53	III
S-6	9.69	1.537	0.47	II
S-7	18.69	1.537	0.76	II
S-8	54.35	0.249	2.81	III
S-9	All covered	0.063	3.54	Ⅰ
S-10	18.69	1.537	0.76	Ⅰ
S-11	18.98	1.537	0.77	Ⅰ
S-12	19.23	1.537	0.79	II
S-13	18.82	1.537	0.77	II
S-14	19.54	1.537	0.8	III
S-15	19.97	1.537	0.85	III
S-16	70.23	1.537	1.94	II
S-17	54.15	1.537	1.79	II
S-18	45.67	1.537	1.42	III
S-19	23.63	1.537	1.33	III
S-20	11.04	1.537	0.69	III
S-21	9.69	1.537	0.51	II

## Data Availability

The data presented in this study are available upon request from the corresponding author. The data are not publicly available due to the funders’ policy.
